# Effectiveness and safety of nivolumab in patients with head and neck cancer in Japanese real-world clinical practice: a multicenter retrospective clinical study

**DOI:** 10.1007/s10147-020-01829-0

**Published:** 2020-11-21

**Authors:** Nobuhiro Hanai, Yasushi Shimizu, Shin Kariya, Ryuji Yasumatsu, Tomoya Yokota, Takashi Fujii, Kiyoaki Tsukahara, Masafumi Yoshida, Kenji Hanyu, Tsutomu Ueda, Hitoshi Hirakawa, Shunji Takahashi, Takeharu Ono, Daisuke Sano, Moriyasu Yamauchi, Akihito Watanabe, Koichi Omori, Tomoko Yamazaki, Nobuya Monden, Naomi Kudo, Makoto Arai, Daiju Sakurai, Takahiro Asakage, Issei Doi, Takayuki Yamada, Akihiro Homma

**Affiliations:** 1 Department of Head and Neck Surgery, Aichi Cancer Center Hospital, Nagoya, Japan; 2 Department of Medical Oncology, Faculty of Medicine and Graduate School of Medicine, Hokkaido University, Sapporo, Japan; 3 Department of Otolaryngology, Head and Neck Surgery, Okayama University Hospital, Okayama, Japan; 4 Department of Otolaryngology, Graduate School of Medical Sciences, Kyushu University, Fukuoka, Japan; 5 Division of Gastrointestinal Oncology, Shizuoka Cancer Center, Nagaizumi, Japan; 6 Department of Head and Neck Surgery, Osaka International Cancer Institute, Osaka, Japan; 7 Department of Otorhinolaryngology, Head and Neck Surgery, Tokyo Medical University, Tokyo, Japan; 8 Otorhinolaryngology and Head and Neck Surgery, The University of Tokyo Hospital, Tokyo, Japan; 9 Head and Neck Oncology Center, International University of Health and Welfare, Mita Hospital, Tokyo, Japan; 10 Department of Otorhinolaryngology, Head and Neck Surgery, Hiroshima University Hospital, Hiroshima, Japan; 11 Department of Otorhinolaryngology, Head and Neck Surgery, University of the Ryukyu Hospital, Nishihara, Japan; 12 Department of Medical Oncology, The Cancer Institute Hospital of Japanese Foundation for Cancer Research, Tokyo, Japan; 13 Department of Otolaryngology, Head and Neck Surgery, Kurume University Hospital, Kurume, Japan; 14 Department of Otolaryngology, Head and Neck Surgery, Yokohama City University Hospital, Yokohama, Japan; 15 Department of Otolaryngology, Head and Neck Surgery, Saga University Hospital, Saga, Japan; 16 Department of Otolaryngology, Head and Neck Surgery, Keiyukai Sapporo Hospital, Sapporo, Japan; 17 Department of Otolaryngology, Head and Neck Surgery, Kyoto University Hospital, Kyoto, Japan; 18 Division of Head and Neck Cancer Oncology, Miyagi Cancer Center, Sendai, Japan; 19 Department of Head and Neck Surgery, National Hospital Organization Shikoku Cancer Center, Matsuyama, Japan; 20 Department of Otorhinolaryngology, Hirosaki University Graduate School of Medicine, Hirosaki, Japan; 21 Department of Medical Oncology, Chiba University Hospital, Chiba, Japan; 22 Department of Otorhinolaryngology, Head and Neck Surgery, Chiba University Hospital, Chiba, Japan; 23 Department of Head and Neck Surgery, Tokyo Medical and Dental University Medical Hospital, Tokyo, Japan; 24 Medical Affairs, ONO Pharmaceutical Co., Ltd, Osaka, Japan; 25 Japan Medical and Development, Bristol-Myers Squibb K.K., Tokyo, Japan; 26 Department of Otolaryngology, Head and Neck Surgery, Faculty of Medicine and Graduate School of Medicine, Hokkaido University, Kita15 Nishi7, Kita-Ku, Sapporo, Hokkaido 060-8638 Japan

**Keywords:** Nivolumab, Real-world clinical practice, Recurrent or metastatic head and neck cancer, Multicenter retrospective study

## Abstract

**Background:**

To fill the data gap between clinical trials and real-world settings, this study assessed the overall effectiveness and safety of nivolumab in patients with head and neck cancer (HNC) during Japanese real-world clinical practice.

**Methods:**

This was a multicenter, retrospective study in Japanese patients with recurrent or metastatic HNC who received nivolumab for the first time between July and December 2017. Data on the clinical use, effectiveness, and safety of nivolumab were extracted from patient medical records.

**Results:**

Overall, 256 patients were enrolled in this study. The median duration of nivolumab treatment was 72.5 days, with patients receiving a median of 6.0 (range 1–27) doses. Median overall survival (OS) was 9.5 (95% confidence interval [CI] 8.2–12.0) months and the estimated 12-month OS rate was 43.2%. The objective response rate (ORR) was 15.7% overall and 21.1%, 7.1%, and 13.6% in patients with primary nasopharynx, maxillary sinus, and salivary gland tumors, respectively, who had been excluded from CheckMate 141. Grade ≥ 3 immune-related adverse events occurred in 5.9% of patients. No new safety signals were identified compared with adverse events noted in CheckMate 141.

**Conclusions:**

The effectiveness and safety of nivolumab in real-world clinical practice are consistent with data from the CheckMate 141 clinical trial. Therapeutic response was also observed in the groups of patients excluded from CheckMate 141.

**Trial registration number:**

UMIN-CTR (UMIN000032600), Clinicaltrials.gov (NCT03569436)

**Electronic supplementary material:**

The online version of this article (10.1007/s10147-020-01829-0) contains supplementary material, which is available to authorized users.

## Introduction

Nivolumab is a fully human immunoglobulin G4 monoclonal antibody targeted against programmed cell death protein-1 (PD-1). Nivolumab was approved in March 2017 for the treatment of recurrent or distant metastatic head and neck cancer (HNC) in Japan. This approval was based on the survival benefits and the manageable safety profile demonstrated by nivolumab in the global phase III CheckMate 141 study, also known as ONO-4538-11 [1]. CheckMate 141 showed that nivolumab significantly prolonged overall survival (OS) compared with standard therapy alone in patients with recurrent squamous cell HNC (median OS of 7.5 vs 5.1 months; *p* = 0.01) [1]. In addition, OS benefit was maintained with 2-year follow-up [2].

In general, patients eligible for clinical trials are highly selected [3]. To be eligible for CheckMate 141, patients were required to have platinum-refractory squamous cell HNC. CheckMate 141 also excluded patients with Eastern Cooperative Oncology Group performance status (ECOG PS) ≥ 2. In addition, the CheckMate 141 study specifically excluded patients with primary tumor sites such as the nasopharynx, nasal cavity, paranasal sinuses, salivary glands, or lip [1].

In melanoma and non-small cell lung cancer, nivolumab has shown long-term survival in patients with stable disease (SD) as well as complete response (CR) or partial response (PR) [4, 5]. In HNC patients, however, the effectiveness by the best overall response (BOR) has not been reported in real-world practice.

In Japan, nivolumab is indicated for any type of HNC, and is not restricted to only those subtypes included in the CheckMate 141 study. In addition, only 18 patients from Japan received nivolumab in CheckMate 141. This means that there is a data gap between the results of the clinical trial and how nivolumab performs in real-world clinical practice in Japan.

The aim of this observational study was to evaluate the effectiveness and safety of nivolumab in a large real-world setting in Japan, and to address the data gap between clinical trial and real-world settings. Here, we report the primary results of a retrospective chart review of patients with HNC who were treated with nivolumab in Japan.

## Patients and methods

### Study design and patients

This was a multicenter, non-interventional, retrospective study conducted at 23 centers in Japan in accordance with relevant regulations in Japan (Ministerial Ordinance on Good Post-Marketing Study Practice, Ministry of Health, Labour and Welfare Ordinance Number 171, December 20, 2004). The study protocol was reviewed and approved by the Institutional Review Board/Independent Ethics Committee at each study site, and the study was conducted according to the ethical principles of the Declaration of Helsinki. Although informed consent was not obtained, patients were given the opportunity to decline to have their clinical records used for research (opt-out consent provision). This study was registered at Clinicaltrials.gov (NCT03569436).

The study includes patients with recurrent or metastatic (distant sites) HNC cancer who were treated with nivolumab for the first time between July 1, 2017, and December 31, 2017. All eligible patients were included except those who had participated in a clinical trial with antineoplastic therapy.

The data were extracted from patients’ medical charts into a specific electronic case report form. Data were collected from baseline until the most recent patient visit. The data cut-off date was 1 year after the first treatment of nivolumab in each patient. Baseline was defined as the visit prior to the start of nivolumab therapy, but the chart review encompassed the period from the diagnosis of HNC to collect data on therapies received prior to nivolumab.

### Endpoints

The primary objectives were to determine the overall effectiveness, including BOR, progression-free survival (PFS), and OS, and to evaluate immune-related adverse events (AEs) in real-world clinical practice. Progression and response primarily recorded by physicians were assessed according to investigator-assessment Response Evaluation Criteria in Solid Tumors (RECIST) 1.1 criteria [6]. Evaluation time was not set due to the nature of this study.

AEs were classified according to the International Council for Harmonisation of Technical Requirements for Pharmaceuticals for Human Use Medical Dictionary for Regulatory Activities Japanese edition (MedDRA/J) Version 21.0.

Drug use information was collected by recording the doses of nivolumab that the patient received, the treatments received before and after administration of nivolumab and their outcomes, the duration of nivolumab treatment, the line of therapy in which patients received nivolumab, any changes in dose or dose interruptions made as a result of AEs, and eventual reason for discontinuation of nivolumab. Platinum-refractory disease in the context of primary therapy was defined as cancer progression within 6 months after the last administration of platinum [1]. Platinum-sensitive disease was defined as cancer progression from 6 months or longer after the last administration of platinum [1].

### Statistical analysis

Effectiveness and safety analyses were performed with all patients who had received ≥ 1 dose of nivolumab. Demographic and baseline characteristics, response data, and AEs were summarized using descriptive statistics (number of patients, mean and standard deviation) for continuous efficacy variables, and frequency and percentage for categorical variables. OS and PFS were estimated and plotted using the Kaplan–Meier method and expressed as the proportion of patients who survived to a specific point in time and median duration, with the corresponding two-sided 95% confidence intervals (CI). For subgroup analyses, tests of statistical significance were conducted using the log-rank test.

Statistical analyses were conducted using SAS Version 9.4 (SAS Institute, Japan).

## Results

### Patient disposition and characteristics

Among 256 registered patients, 79% were men and the median age was 66 years (range 20–84 years; Table 1). Of the 246 patients with known ECOG PS score, 31 patients (12.6%) had a performance status of ≥ 2. Overall, 198 of the 239 patients (82.8%) with known disease stage had stage III or IV disease at the time of HNC diagnosis (Table 1). Most patients (*n* = 217; 84.8%) had squamous cell carcinoma, but 29 patients (11.3%) had non-squamous cell histology. Primary tumor sites were the hypopharynx (*n* = 64; 25.0%), oral cavity (*n* = 56; 21.9%), oropharynx (*n* = 40; 15.6%), salivary gland (*n* = 23; 9.0%), larynx (*n* = 21; 8.2%), nasopharynx (*n* = 19; 7.4%), maxillary sinus (*n* = 14; 5.5%), and other sites (*n* = 19; 7.4%).

**Table 1:** Baseline characteristics of patients

Characteristics	Nivolumab (*N* = 256)
Sex, *n* (%)	
Male	202 (78.9)
Female	54 (21.1)
Age, years	
Median (range)	66 (20–84)
Age ≥ 75 years, *n* (%)	24 (9.4)
Primary cancer site, *n* (%)	
Hypopharynx	64 (25.0)
Oral cavity	56 (21.9)
Oropharynx	40 (15.6)
Salivary glands*	23 (9.0)
Larynx	21 (8.2)
Nasopharynx*	19 (7.4)
Maxillary sinus*	14 (5.5)
Others*	19 (7.4)
Presence of another primary malignancy, *n* (%)	
Yes*	49 (19.1)
No	199 (77.7)
Unknown	8 (3.1)
Head and neck cancer histology, *n* (%)	
Squamous cell carcinoma	217 (84.8)
Non-Squamous cell carcinoma*	29 (11.3)
Not Evaluated*	9 (3.5)
ECOG PS, *n* (%)	
0	118 (46.1)
1	97 (37.9)
2*	23 (9.0)
3*	6 (2.3)
4*	2 (0.8)
Unknown*	10 (3.9)
Cancer stage at nivolumab initiation, *n* (%)	
I*	11 (4.3)
II*	30 (11.7)
III	22 (8.6)
IVA	114 (44.5)
IVB	29 (11.3)
IVC	33 (12.9)
Unknown*	17 (6.6)
Nivolumab treatment line for recurrent or metastatic head and neck cancer, *n* (%)**	
1st	70 (27.3)
2nd	110 (43.0)
3rd	45 (17.6)
4th or later	31 (12.1)

*ECOG* Eastern Cooperative Oncology Group

^*^Patients with these characteristics were excluded from the CheckMate 141 study [1]

^**^Nivolumab treatment line was counted as the number of systemic chemotherapy for recurrent or metastatic HNC

Seventy patients (27.3%) received nivolumab as the first-line treatment for recurrent/metastatic HNC, 110 (43.0%) as second-line, and 76 (29.7%) as third-line or later treatment.

After the 12-month follow-up, 14.1% (*n* = 36) were still under the treatment with nivolumab. Of the 220 patients (85.9%) who completed nivolumab treatment, 170 patients (77.3%) had shown disease progression and nine patients had died. The median (min, max) duration of nivolumab treatment was 72.5 (1, 380) days, with patients receiving a median of 6.0 (range 1–27) doses of nivolumab (Table S1).

### Overall effectiveness

**Fig. 1:**
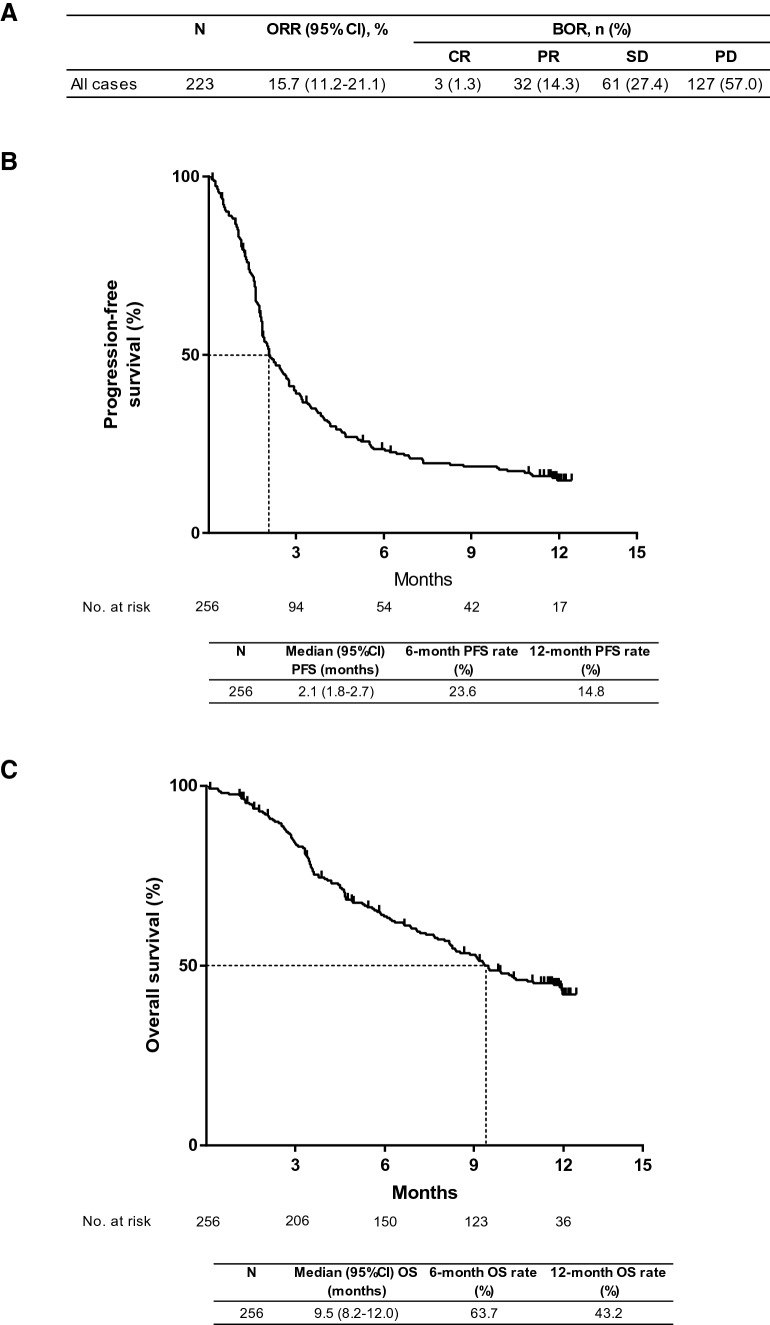
**a** Overall response rate **b** progression-free survival and **c** overall survival among all patients. *CI* confidence intervals, *BOR* best overall response, *CR* complete response, *ORR* objective response rate, *OS* overall survival, *PD* progressive disease, *PFS* progression-free survival, *PR* partial response, *SD* stable disease

The BOR was assessed in 223 of 256 evaluable patients. Among these 223 patients, the BOR was CR in 3 patients (1.3%) and PR in 32 patients (14.3%), for an objective response rate (ORR) of 15.7 (95% CI 11.2–21.1) % (*n* = 35). An additional 61 patients (27.4%) had SD, resulting in a disease control rate of 43.0% (Fig. 1a). The median duration of response was 5.1 (95% CI 2.8–NE) months. The median PFS was 2.1 (95% CI 1.8–2.7) months (Fig. 1b) and the median OS in the 256 patients treated with nivolumab was 9.5 (95% CI 8.2–12.0) months (Fig. 1c). The estimated 12-month OS rate was 43.2 (95% CI 36.7–49.5)%, with an estimated 12-month PFS rate of 14.8 (95% CI 10.5–19.7)%.

### Effectiveness by subgroup

**Fig. 2:**
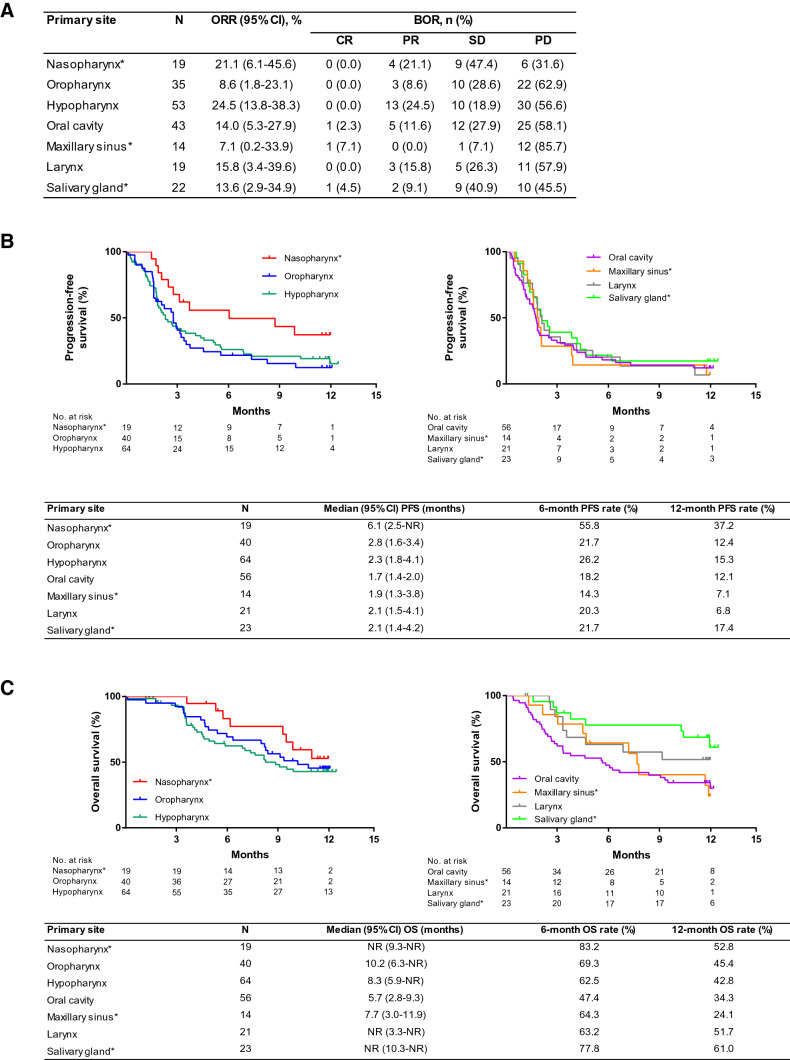
**a** Overall response rate **b** progression-free survival and **c** overall survival among patients stratified according to the primary site. *BOR* best overall response, *CR* complete response, *NR* not reached, *ORR* objective response rate, *OS* overall survival, *PD* progressive disease, *PFS* progression-free survival, *PR* partial response, *SD* stable disease. *Primary tumor types excluded from Checkmate 141 study

Regarding the effectiveness of nivolumab in Japanese patients with primary tumor sites specifically excluded in the CheckMate 141 study, the ORR was 21.1 (95% CI 6.1–45.6) %, 13.6 (95% CI 2.9–34.9) %, and 7.1 (95% CI 0.2–33.9) % in the nasopharynx, salivary glands, and maxillary sinus, respectively (Fig. 2a). The median PFS was 6.1 (95% CI 2.5–NE) months in the nasopharynx, 2.1 (95% CI 1.4–4.2) months in the salivary glands, and 1.9 (95% CI 1.3–3.8) months in the maxillary sinus with 12-month PFS rates of 37.2%, 17.4%, and 7.1% for the nasopharynx, salivary glands, and maxillary sinus, respectively (Fig. 2b). Finally, the median OS was not reached for the nasopharynx and salivary glands and was 7.7 (95% CI 3.0–11.9) months in the maxillary sinus (Fig. 2c). Further, the 12-month OS rate in the nasopharynx, salivary glands, and maxillary sinus was 52.8%, 61.0%, and 24.1%, respectively. This study included a small number of patients (*n* = 29) with non-squamous cell carcinoma (non-SCC) in addition to patients with SCC (*n* = 217). Effectiveness according to histological type (SCC versus non-SCC) is shown in Figure S1. ORR, PFS, and OS were similar between patients with SCC or non-SCC with no statistically significant differences noted for any comparison. The primary site and histology of patients with non-SCC are detailed in Table S2.

**Fig. 3:**
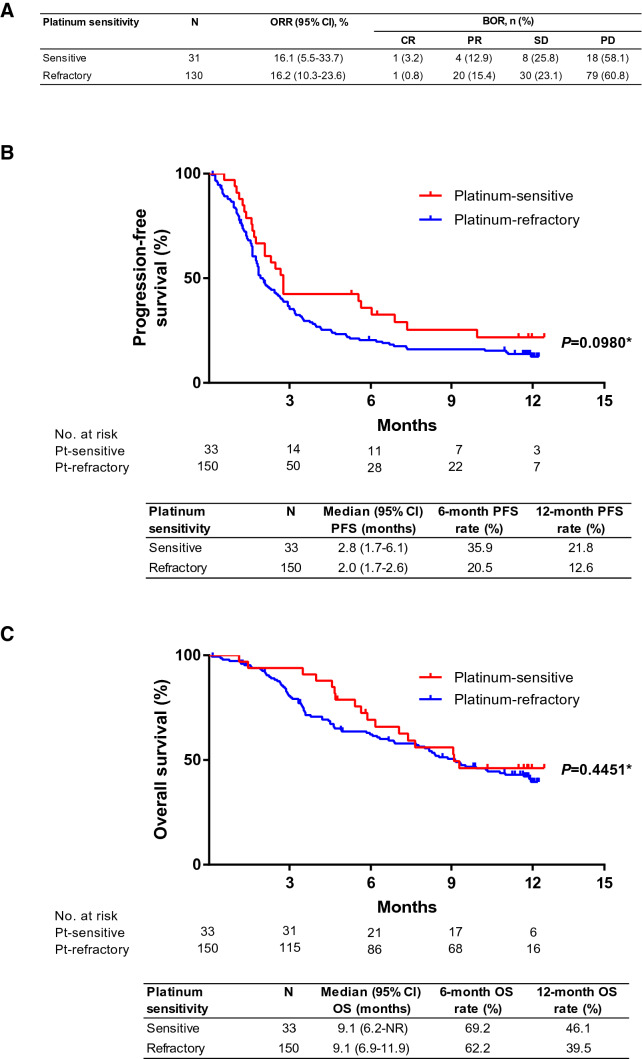
**a** Overall response rate **b** progression-free survival and **c** overall survival among patients stratified according to platinum-sensitive or platinum-refractory status. *BOR* best overall response, *CR* complete response, *ORR* objective response rate, *OS* overall survival, *PD* progressive disease, *PFS* progression-free survival, *PR* partial response, *SD* stable disease. *Log-rank test

In the present real-world study, nivolumab use was not limited to platinum-refractory but also platinum-sensitive patients. In a subgroup analysis by prior platinum responsiveness, the ORR was 16.2 (95% CI 10.3–23.6)% and 16.1 (95% CI 5.5–33.7) % in platinum-refractory patients and platinum-sensitive patients, respectively (Fig. 3a). The median PFS was 2.0 (95% CI 1.7–2.6) months in platinum-refractory and 2.8 (95% CI 1.7–6.1) months in platinum-sensitive (Fig. 3b). The median OS was 9.1 (95% CI 6.9–11.9) months and 9.1 (95% CI 6.2–NR) months for platinum-refractory and platinum-sensitive patients, respectively (Fig. 3c).

**Fig. 4:**
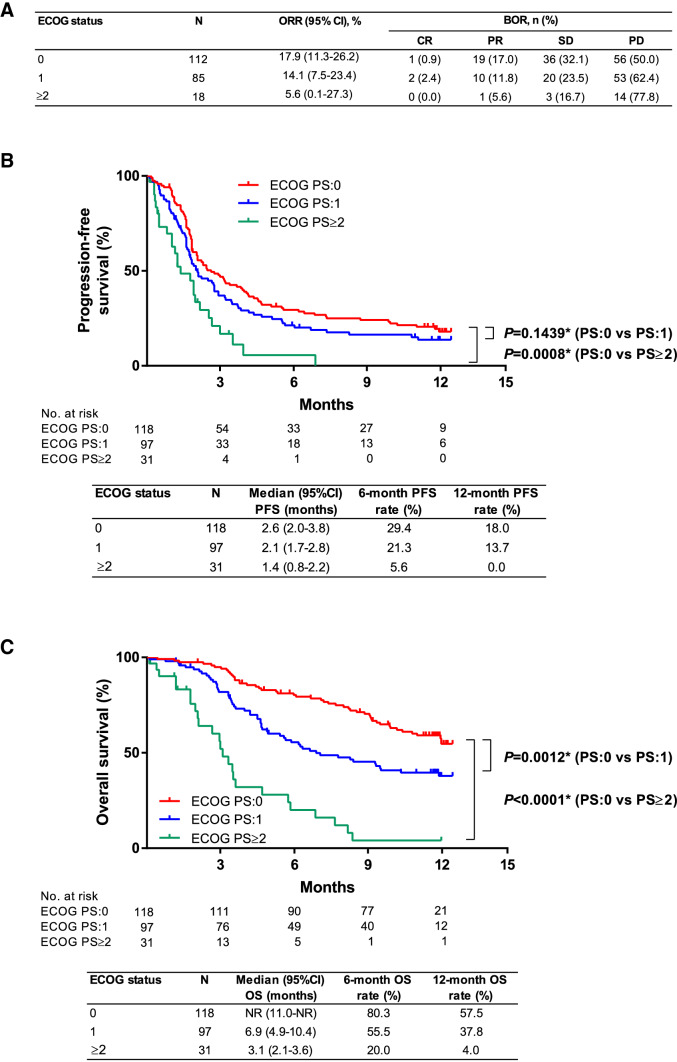
**a** Overall response rate **b** progression-free survival and **c** overall survival among patients according to ECOG status. *BOR* best overall response, *CR* complete response, *ECOG PS* Eastern Cooperative Oncology Group performance status, *NR* not reached, *ORR* objective response rate, *OS* overall survival, *PD* progressive disease, *PFS* progression-free survival, *PR* partial response, *SD* stable disease. *Log-rank test

In a subgroup analysis by PS, the ORR, PFS, and OS were generally numerically greater in patients with ECOG PS 0 status, compared with patients with ECOG PS 1 or, in particular, ECOG PS ≥ 2 status (Fig. 4). The ORR was 17.9 (95% CI 11.3–26.2) %, 14.1 (95% CI 7.5–23.4) %, and 5.6 (95% CI 0.1–27.3) % in patients with ECOG PS 0, ECOG PS 1, and ECOG PS ≥ 2 status, respectively (Fig. 4a). PFS was 2.6 (95% CI 2.0–3.8) months, 2.1 (95% CI 1.7–2.8) months, and 1.4 (95% CI 0.8–2.2) months in patients with ECOG PS 0, ECOG PS 1, and ECOG PS ≥ 2 status, respectively (Fig. 4b). The median OS was not reached in patients with ECOG PS 0 status and was 6.9 (95% CI 4.9–10.4) months and 3.1 (95% CI 2.1–3.6) months in patients with ECOG PS 1 and ECOG PS ≥ 2 status, respectively (Fig. 4c).

**Fig. 5:**
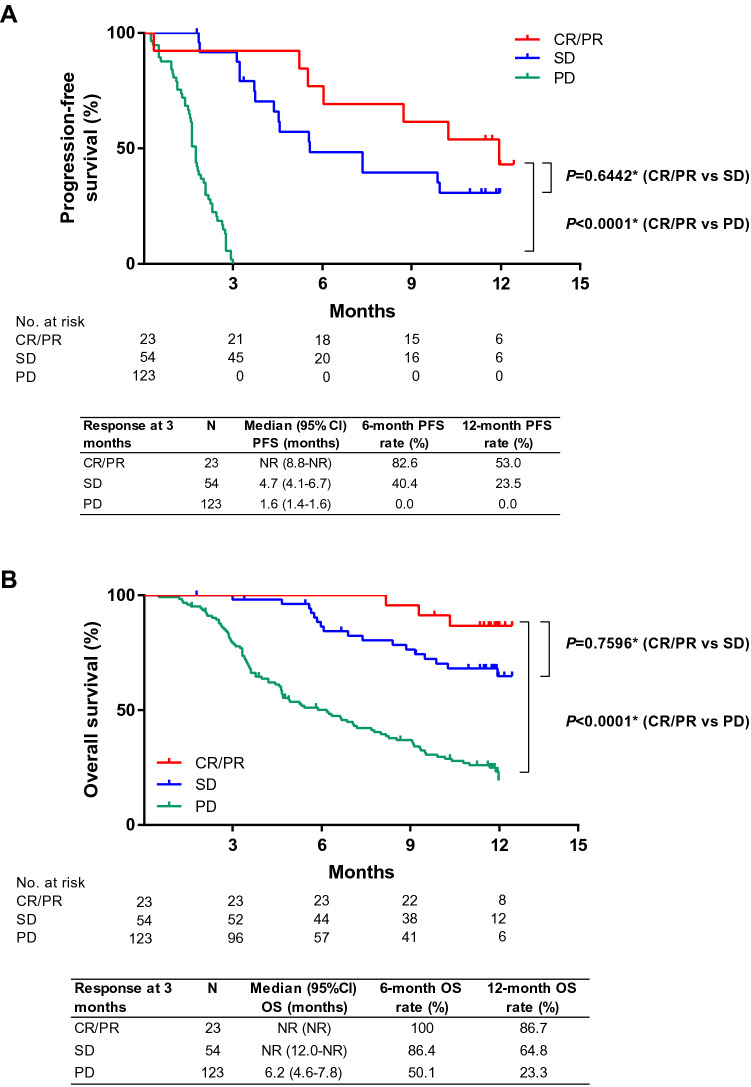
**a** Progression-free survival and **b** overall survival according to the best overall response at 3 months. *CR* complete response, *OS* overall survival, *PD* progressive disease, *PFS* progression-free survival, *PR* partial response, *SD* stable disease. *Log-rank test

Effectiveness by BOR at 3 months are shown in Fig. 5. Both PFS and OS were greater in patients with CR/PR or SD compared with patients who experienced progressive disease (Fig. 5a, b). Median PFS was not reached in patients with CR/PR and was 4.7 (95% CI 4.1–6.7) months and 1.6 (95% CI 1.4–1.6) months in patients with SD and PD, respectively (Fig. 5a). Median OS was not reached in patients with SD or CR/PR at 3 months and 6.2 (95% CI 4.6–7.8) months in patients with progressive disease (Fig. 5b).

### Incidence of AEs

Thirty-eight patients (14.8%) developed immune-related AEs of any grade; 15 patients (5.9%) developed a grade ≥ 3 immune-related AE (Table 2). Endocrine disorders were the most common type of immune-related AEs which affected 14 patients, but only two of these events were grade ≥ 3. The most common type of grade ≥ 3 immune-related AEs were lung disorders (*n* = 6). One patient developed a grade 5 immune-related AE (interstitial pneumonia). The median time to onset of any immune-related AEs was 8.7 (0.1–43.7) weeks; each categorized immune-related AE widely appeared throughout the 12-month observation periods. Median time to resolution of most immune-related AEs was 3 to 6 weeks.

**Table 2:** Incidence of immune-related adverse events over 12 months

Immune-related AE category	No. of patients, *n* (%)		No. of events			
	Any grade	Grade ≥ 3	All grade, *n*	Median (range) time to onset, weeks	Resolved, *n* (recovery rate, %)	Median (range) time to resolution, weeks
Any immune-related AE	38 (14.8)	15 (5.9)	47	8.7 (0.1–43.7)	34 (72.3)	4.8 (0–43.4)
Endocrine disorder	14 (5.5)	2 (0.8)	14	9.3 (0.1–43.0)	9 (64.3)	5.1 (1.4–12.0)
Lung disorder	8 (3.1)	6 (2.3)	8	3.0 (0.3–27.3)	4 (50.0)	4.8 (3.0–11.3)
Skin disorder	7 (2.7)	2 (0.8)	8	21.2 (4.0–33.1)	6 (75.0)	2.5 (2.0–22.0)
Liver disorder	6 (2.3)	3 (1.2)	7	12.0 (2.0–29.0)	7 (100.0)	4.7 (0.7–27.3)
Gastrointestinal disorder	3 (1.2)	1 (0.4)	3	9.4 (9.0–43.7)	3 (100.0)	6.4 (0–14.0)
Blood disorder	2 (0.8)	1 (0.4)	2	5.8 (4.0–7.6)	1 (50.0)	43.4 (43.4–43.4)
Other	5 (2.0)	2 (0.8)	5	4.0 (2.0–29.0)	4 (80.0)	3.1 (0–27.0)

Time to onset refers to the time after the first dose of nivolumab and time to resolution refers to time from the onset of the adverse event until complete resolution

*AE* adverse event

## Discussion

To the best of our knowledge, this is the largest study to evaluate the effectiveness and safety of nivolumab in patients with HNC in Japanese real-world clinical practice. Median PFS (2.1 vs 2.0 months), 12-month OS rate (43% vs 36%), and median OS (9.5 vs 7.5 months) in our study were similar to those in CheckMate 141 [1]. Furthermore, our data are also comparable to other reported real-world data in Japan [7, 8].

The current study addresses several data gaps between the clinical trial and real-world settings. First of all, the current study reveals the effectiveness of nivolumab in patients with primary tumors in the nasopharynx, maxillary sinus, or salivary gland, who would have been excluded from previous phase III clinical trials for HNC including the CheckMate 141, KEYNOTE-040, KEYNOTE-048, and EXTREME studies [9–11]. In particular, we found a patient with maxillary sinus cancer responded to nivolumab, which is the first positive result recorded for any checkpoint inhibitor monotherapies in such patients [12]. In addition, the effectiveness of nivolumab for nasopharyngeal and salivary gland tumors in the current study was consistent with the results in prior phase I/II trials [9, 13–15]. Historical data showed that prognosis is generally poor in patients with primary HNC in the hypopharynx, oral cavity, and nasal sinuses, whereas prognosis tends towards improvement in patients with nasopharyngeal or laryngeal primary tumors [10, 16–19]. It has been suggested that the primary HNC site is associated with poor response to chemotherapy [20]. By contrast, the current study showed that patients well responded to nivolumab irrespective of the expected prognosis.

The effectiveness of nivolumab for platinum-refractory HNC in this study was comparable to that of CheckMate 141. While most patients receiving nivolumab in real-world clinical practice have platinum-refractory disease, 18% of the patients in our study had platinum-sensitive disease, a population that was not included in CheckMate 141 [1]. In the present study, ORR and OS were numerically similar between patient populations with platinum-refractory and platinum-sensitive diseases. However, because the effectiveness of the platinum-sensitive disease has been evaluated only in a limited number of patients [9], further large-scale studies are warranted. Stratification of patients by performance status in this study showed that nivolumab was most effective, in terms of ORR, PFS, and OS, in patients with ECOG PS 0 status. Other studies utilizing anti-PD-1 inhibitors, including nivolumab, in various real-world settings including advanced melanoma, non-small cell lung cancer, and head and neck cancer have demonstrated that favorable PS is predictive of greater OS and PFS [21–23]. Recent longitudinal studies of anti-PD-1 inhibitors, also conducted in real-world clinical practice settings with median follow-up periods of up to 12.9 months, further verify better responses in patients with ECOG PS 0–1 status versus those with ECOG PS > 1 status [24–26]. Therefore, the present results provide additional evidence to support the notion that nivolumab and similar agents in this class are more effective in patients who are in good general condition at treatment initiation.

Superior PFS and OS were observed in patients whose BOR at 3 months was CR, PR, or SD. This is consistent with previous findings with nivolumab in patients with melanoma or non-small cell lung cancer [4, 5], and is also consistent with the known response profile to immune-checkpoint inhibitors. In addition, even after tumor progression, half of the patients with PD survived for more than 6 months. These patients may receive subsequent chemotherapy after discontinuation of the nivolumab therapy, which may contribute to prolonged survival because it has been reported for several cancers that chemotherapy after immunotherapy is highly effective [27–30]. Future studies with longer follow-up will reveal possibly different impacts of subsequent chemotherapy.

The incidence and timing of immune-related AEs in our study were similar to or even lower than previous reports, including CheckMate 141 [1, 31], and with Japanese clinical use of nivolumab in patients with HNC [8]. No new safety signals were identified in our study compared with CheckMate 141, despite the fact that our study included a more vulnerable patient population. These data indicate that nivolumab has a well-characterized and manageable safety profile in real-world clinical use.

Retrospective observational studies have limitations. As an observational design, there was no control group. Because completed by individual physicians during real-world clinical practice, patients’ medical records may not always contain complete and comparable information and may contain measurement errors. To recruit a large number of patients, we preferentially included centers that treat a high number of patients with HNC. This may have introduced some selection bias in the study population.

In conclusion, this retrospective observational analysis in a real-world clinical setting supports the effectiveness and safety of nivolumab in a range of Japanese patients with HNC. No new safety signals were identified, and the findings of randomized clinical trials with nivolumab are applicable to a real-world population of patients with a more diverse clinical profile in terms of performance status and primary site.

## Electronic supplementary material

Below is the link to the electronic supplementary material.

Supplementary file1 (DOCX 55 KB)
