# Correction to: Effectiveness and safety of nivolumab in patients with head and neck cancer in Japanese real‑world clinical practice: a multicentre retrospective clinical study

**DOI:** 10.1007/s10147-021-01879-y

**Published:** 2021-04-10

**Authors:** Nobuhiro Hanai, Yasushi Shimizu, Shin Kariya, Ryuji Yasumatsu, Tomoya Yokota, Takashi Fujii, Kiyoaki Tsukahara, Masafumi Yoshida, Kenji Hanyu, Tsutomu Ueda, Hitoshi Hirakawa, Shunji Takahashi, Takeharu Ono, Daisuke Sano, Moriyasu Yamauchi, Akihito Watanabe, Koichi Omori, Tomoko Yamazaki, Nobuya Monden, Naomi Kudo, Makoto Arai, Daiju Sakurai, Takahiro Asakage, Issei Doi, Takayuki Yamada, Akihiro Homma

**Affiliations:** 1 Department of Head and Neck Surgery, Aichi Cancer Center Hospital, Nagoya, Japan; 2 Department of Medical Oncology, Faculty of Medicine and Graduate School of Medicine, Hokkaido University, Sapporo, Japan; 3 Department of Otolaryngology, Head and Neck Surgery, Okayama University Hospital, Okayama, Japan; 4 Department of Otolaryngology, Graduate School of Medical Sciences, Kyushu University, Fukuoka, Japan; 5 Division of Gastrointestinal Oncology, Shizuoka Cancer Center, Nagaizumi, Japan; 6 Department of Head and Neck Surgery, Osaka International Cancer Institute, Osaka, Japan; 7 Department of Otorhinolaryngology, Head and Neck Surgery, Tokyo Medical University, Tokyo, Japan; 8 Otorhinolaryngology and Head and Neck Surgery, The University of Tokyo Hospital, Tokyo, Japan; 9 Head and Neck Oncology Center, International University of Health and Welfare, Mita Hospital, Tokyo, Japan; 10 Department of Otorhinolaryngology, Head and Neck Surgery, Hiroshima University Hospital, Hiroshima, Japan; 11 Department of Otorhinolaryngology, Head and Neck Surgery, University of the Ryukyu Hospital, Nishihara, Japan; 12 Department of Medical Oncology, The Cancer Institute Hospital of Japanese Foundation for Cancer Research, Tokyo, Japan; 13 Department of Otolaryngology, Head and Neck Surgery, Kurume University Hospital, Kurume, Japan; 14 Department of Otolaryngology, Head and Neck Surgery, Yokohama City University Hospital, Yokohama, Japan; 15 Department of Otolaryngology, Head and Neck Surgery, Saga University Hospital, Saga, Japan; 16 Department of Otolaryngology, Head and Neck Surgery, Keiyukai Sapporo Hospital, Sapporo, Japan; 17 Department of Otolaryngology, Head and Neck Surgery, Kyoto University Hospital, Kyoto, Japan; 18 Division of Head and Neck Cancer Oncology, Miyagi Cancer Center, Sendai, Japan; 19 Department of Head and Neck Surgery, National Hospital Organization Shikoku Cancer Center, Matsuyama, Japan; 20 Department of Otorhinolaryngology, Hirosaki University Graduate School of Medicine, Hirosaki, Japan; 21 Department of Medical Oncology, Chiba University Hospital, Chiba, Japan; 22 Department of Otorhinolaryngology, Head and Neck Surgery, Chiba University Hospital, Chiba, Japan; 23 Department of Head and Neck Surgery, Tokyo Medical and Dental University Medical Hospital, Tokyo, Japan; 24 Medical Affairs, ONO Pharmaceutical Co., Ltd, Osaka, Japan; 25 Japan Medical and Development, Bristol-Myers Squibb K.K., Tokyo, Japan; 26 Department of Otolaryngology, Head and Neck Surgery, Faculty of Medicine and Graduate School of Medicine, Hokkaido University, Kita15 Nishi7, Kita-Ku, Sapporo, Hokkaido 060-8638 Japan

## Correction to: International Journal of Clinical Oncology 10.1007/s10147-020-01829-0

The article Effectiveness and safety of nivolumab in patients with head and neck cancer in Japanese real‑world clinical practice: a multicentre retrospective clinical study, written by Nobuhiro Hanai, Yasushi Shimizu, Shin Kariya, Ryuji Yasumatsu, Tomoya Yokota, Takashi Fujii, Kiyoaki Tsukahara, Masafumi Yoshida, Kenji Hanyu, Tsutomu Ueda, Hitoshi Hirakawa, Shunji Takahashi, Takeharu Ono, Daisuke Sano, Moriyasu Yamauchi, Akihito Watanabe, Koichi Omori, Tomoko Yamazaki, Nobuya Monden, Naomi Kudo, Makoto Arai, Daiju Sakurai, Takahiro Asakage, Issei Doi, Takayuki Yamada and Akihiro Homma, was originally published Online First without Open Access. With the author(s)’ decision to opt for Open Choice the copyright of the article changed on January 18, 2021 to © Author(s) 2021and the article is forthwith distributed under a Creative Commons Attribution 4.0 International License, which permits use, sharing, adaptation, distribution and reproduction in any medium or format, as long as you give appropriate credit to the original author(s) and the source, provide a link to the Creative Commons licence, and indicate if changes were made. The images or other third party material in this article are included in the article’s Creative Commons licence, unless indicated otherwise in a credit line to the material. If material is not included in the article’s Creative Commons licence and your intended use is not permitted by statutory regulation or exceeds the permitted use, you will need to obtain permission directly from the copyright holder. To view a copy of this licence, visit http://creativecommons.org/licenses/by/4.0.

The Author also likes to revise the following in the Original publication as below:

In the section of "Incidence of AE" The median time to onset of any immune-related AEs was 8.7 (0.1 −43.7) weeks; (months should be weeks).

The original article has been corrected.

