# Supplementary material for: Effectiveness and safety of nivolumab in patients with head and neck cancer in Japanese real-world clinical practice: a multicenter retrospective clinical study
Source: Int J Clin Oncol. 2020 Nov 21;26(3):494–506. doi: 10.1007/s10147-020-01829-0 (PMC7895797; doi:10.1007/s10147-020-01829-0)

# Supplementary Material

**Effectiveness and safety of nivolumab in patients with head and neck cancer in Japanese real-world clinical practice: A multicenter retrospective clinical study**

**The International Journal of Clinical Oncology**

Nobuhiro Hanai, Yasushi Shimizu, Shin Kariya, Ryuji Yasumatsu, Tomoya Yokota, Takashi Fujii, Kiyoaki Tsukahara, Masafumi Yoshida, Kenji Hanyu, Tsutomu Ueda, Hitoshi Hirakawa, Shunji Takahashi, Takeharu Ono, Daisuke Sano, Moriyasu Yamauchi, Akihito Watanabe, Koichi Omori, Tomoko Yamazaki, Nobuya Monden, Naomi Kudo, Makoto Arai, Daiju Sakurai, Takahiro Asakage, Issei Doi, Takayuki Yamada, Akihiro Homma

**Corresponding Author:** Akihiro Homma, E-mail: [ak-homma@med.hokudai.ac.jp](mailto:ak-homma@med.hokudai.ac.jp)

**Table S1.** Nivolumab treatment parameters

|  | **Nivolumab** |
| --- | --- |
| Line of treatment |  |
| Median (range) | 2.0 (1–6) |
| IQR | 1.0–3.0 |
| Mean (SD) | 2.2 (1.1) |
| Number of doses |  |
| Median (range) | 6.0 (1–27) |
| IQR | 3.0–12.0 |
| Mean (SD) | 8.9 (7.8) |
| Duration of administration (days) |  |
| Median (range) | 72.5 (1–380) |
| IQR | 31.5–173.0 |
| Mean (SD) | 122.9 (120.2) |

IQR, interquartile range; SD, standard deviation

**Table S2.** Primary site and histology of patients with non-squamous cell carcinoma

| **Primary site** | **Histology** | **No. of cases** |
| --- | --- | --- |
| Salivary gland | Salivary duct cancer | 7 |
|  | Adenocarcinoma | 2 |
|  | Adenocarcinoma (not otherwise specified) | 2 |
|  | Mucoepidermoid carcinoma | 2 |
|  | Parotid gland cancer | 1 |
|  | Parotid duct cancer | 1 |
|  | Adenoid cystic carcinoma | 1 |
|  | Poorly differentiated carcinoma | 1 |
| Maxillary sinus | Myoepithelioma | 1 |
|  | Neuroendocrine carcinoma | 1 |
|  | Adenoid cystic carcinoma | 1 |
| Hypopharynx | Lymphoepithelioma | 1 |
|  | Neuroendocrine carcinoma | 1 |
| Oropharyngeal | Adenoid cystic carcinoma | 1 |
|  | Salivary duct cancer | 1 |
| Other | Myoepithelioma | 1 |
|  | Adenocarcinoma | 1 |
|  | Adenoid cystic carcinoma (unknown primary site) | 1 |
|  | Undifferentiated carcinoma | 1 |
|  | Olfactory neuroblastoma | 1 |

**Figure S1.** (A) Overall response rate (B) progression-free survival and (C) overall survival among patients according to histology status. BOR, best overall response; CR, complete response; ECOG PS, Eastern Cooperative Oncology Group performance status; NR, not reached; ORR, objective response rate; OS, overall survival; PD, progressive disease; PFS, progression-free survival; PR, partial response; SCC, squamous cell carcinoma; SD, stable disease. *log-rank test


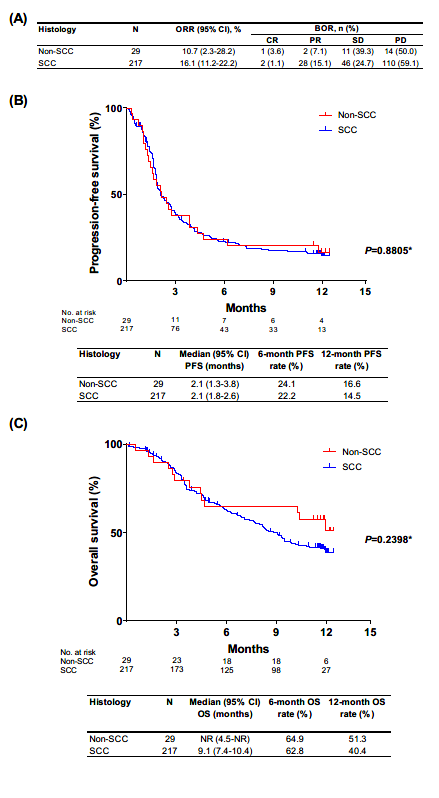

Supplement: Supplementary file 1 — Supplementary file1 (DOCX 55 KB) [file 10147_2020_1829_MOESM1_ESM.docx]
